# Valorization of Food By-Products into a Zero-Waste Broth: Effects of Yeast Extract on Composition and Sensory Properties

**DOI:** 10.3390/molecules31091529

**Published:** 2026-05-05

**Authors:** Anna Otlewska, Katarzyna Dybka-Stępień, Katarzyna Rajkowska, Anna Koziróg, Agnieszka Nowak, Małgorzata Piotrowska, Aleksandra Czerbniak-Włodarczyk, Agata Czyżowska, Joanna Grzelczyk, Anna Kołczyk

**Affiliations:** 1 Institute of Fermentation Technology and Microbiology, Faculty of Biotechnology and Food Sciences, Lodz University of Technology, Wolczanska 171/173, 90-530 Lodz, Poland; anna.otlewska@p.lodz.pl (A.O.); katarzyna.dybka@p.lodz.pl (K.D.-S.); anna.kozirog@p.lodz.pl (A.K.); agnieszka.nowak@p.lodz.pl (A.N.); malgorzata.piotrowska@p.lodz.pl (M.P.); agata.czyzowska@p.lodz.pl (A.C.); 2 Institute of Food Technology and Analysis, Faculty of Biotechnology and Food Sciences, Lodz University of Technology, Stefanowskiego 2/22, 90-537 Lodz, Poland; joanna.grzelczyk@p.lodz.pl; 3 FoodHub S.A., Wielunska 2, 97-438 Rusiec, Poland; anna.kolczyk@foodhub.com.pl

**Keywords:** by-product utilization, nutritional composition, antioxidant activity, bioactive compounds, hedonic testing, flavor enhancer

## Abstract

Food losses and waste occur throughout the food production chain, creating an urgent need for their recovery and valorization into sustainable food products. Sensory acceptance is crucial for consumer interest and market success and can be enhanced using yeast extract. However, strategies that simultaneously valorize meat and vegetable by-products into nutritionally and functionally enriched products while ensuring sensory acceptability have been scarcely investigated. This study examined whether poultry carcasses after mechanical meat separation, combined with root vegetable processing residues, could be converted into a zero-waste broth with enhanced nutritional and bioactive-related properties. The developed broth was obtained by cooking for 3 h at 122 °C under 0.2 MPa, with 2% (*v*/*v*) yeast extract added to improve umami taste. The broth was analyzed for amino acids, protein and nucleoside content, turbidity, vitamins A, K, and B-group, and antioxidant activity. The yeast-extract-enriched broth showed high levels of these vitamins, supporting EU-compliant nutrition and health claims, and demonstrated strong antioxidant activity as measured by in vitro assays. Preliminary sensory testing indicated high acceptability of the broth, likely due to the addition of yeast extract. Overall, the study demonstrates that meat and vegetable by-products can be effectively valorized into a nutritionally enriched and compositionally promising product.

## 1. Introduction

Meals should not only supply consumers with the necessary nutrients and calories from the ingredients but also offer an attractive taste. Food should be palatable to increase the pleasure derived from its consumption. In savory dishes, including soups, the attractiveness of the flavor is often associated with the presence of umami taste as well as the impression of “depth” and “richness” of the flavor associated with kokumi [1,2].

Today, consumers pay increasing attention to the ingredient composition of the products they buy and consume. This also applies to food, especially ready-to-eat meals, including soups. Soup is an important category of meal in the European market, including the Polish market. Soup remains popular amongst consumers, and the European soup market size was estimated at $3 billion in 2024 and is expected to reach $3.8 billion by 2033 [3]. According to Statista [4], revenue in the soup market in Poland is expected to grow at an annual growth rate (CAGR 2025-2030) of 5.64%. As in all of Europe, as well as in Poland, the role of soup is shifting from a traditional, home-cooked dish to a convenient meal option that balances taste, ease of preparation, and health. There is a growing interest in functional, natural, and plant-based options to fit modern lifestyles.

The zero-waste food sector demonstrates promising opportunities for expansion, as 85% of consumers in the United Kingdom report a willingness to purchase foods and beverages made with upcycled ingredients. Likewise, a study by Coderoni and Perito [5] from Italy showed that 56% of participants would buy products manufactured from food waste and by-products, and when such zero-waste products were additionally linked to lower environmental impacts during production, consumer acceptance increased to 69% [5,6]. In responsible, sustainable industrial food production, in line with the principles of a zero-waste economy, every aspect is of paramount importance. The production process should be thoroughly considered to ensure maximum use of raw materials, limit the production of by-products and reuse them effectively, reduce packaging, and minimize losses in the storage of prepared meals. Efficient resource management, including the use of by-products from other processes and the use of local suppliers, is a conscious and responsible approach by food producers towards both consumers and the natural environment, significantly reducing food waste, which has become a problem, especially in highly developed countries.

An example of this trend is the rising popularity of “zero-waste” soups made with natural food ingredients that can replace chemical flavor enhancers, which are now often viewed negatively. Monosodium glutamate (MSG) is one such ingredient that is considered a controversial additive. As a result, soup manufacturers are increasingly removing MSG from recipes in response to consumer preferences for “clean label” and natural ingredient lists. Recently, in the context of developing natural flavor enhancers, substances naturally rich in umami and kokumi components have been considered promising alternatives to MSG addition in food. Thus, MSG substitutes such as tomato concentrates, mushroom concentrates, and yeast extracts are gaining increasing attention [7]. Food manufacturers use the synergistic umami effect instead of adding MSG. The synergistic umami effect results from the combination of naturally occurring glutamate and 5′-monophosphate nucleotides (a mixture of 5′-inosine monophosphate and 5′-guanosine monophosphate). This synergistic effect is commonly known from its presence in food such as cheese, seaweed, fish sauce, dashi (which combines inosinic acid from bonito fish with kombu seaweed—a natural source of glutamic acid) and other broths (like traditional European chicken broth), soy sauce, fermented soybean, yeast extract, and dried mushrooms, to name a few. Employing this umami and kokumi synergy enhances both the flavor and the perception of food taste and complexity, as well as the product’s health profile. Such a combination also encourages greater consumption of vegetables, mushrooms, fish, and poultry while helping reduce intake of high-fat foods and salt [8,9,10]. To achieve a pronounced umami taste, approximately 0.4% of MSG should be added to food. At the same time, a twenty-time lower amount of 5′-monophosphate nucleotides added to food will result in the same flavor enhancement [10,11].

Yeast and yeast-derived products have been used in the food industry for centuries (e.g., in bread, beer, and wine production) as well as in animal feed, pharmaceutical, or cosmetic applications. Several yeast species, including *Saccharomyces cerevisiae*, *Candida utilis*, *Kluyveromyces marxianus*, and *Yarrowia lipolytica*, are recognized as safe and possess GRAS (Generally Recognized As Safe) status granted by the United States Food and Drug Administration and/or QPS (Qualified Presumption of Safety) by the European Food Safety Authority [12,13]. Yeast extracts are also classified as GRAS and considered safe for consumers. They can serve as natural enhancers in food products due to their content of umami- and kokumi-enhancing compounds. Yeast extracts contain glutamic acid and 5′-nucleotides (particularly 5′-GMP and 5′-IMP) as well as valuable proteins, peptides, amino acids, carbohydrates, vitamins (group B), and minerals. Their composition gives them an advantageous nutritional profile suitable for consumption [14]. What is more, yeast extract can effectively mask the absence of salt in various culinary applications, supporting the development of healthier meals [14,15,16]. However, the profile of chemical compounds in yeast extracts can vary significantly depending on several factors, such as yeast species, cultivation conditions, the yeast extract production method, and degree of hydrolysis [15].

In this work, we aimed to repurpose meat processing by-products, which are poultry carcasses, along with vegetable waste processing, i.e., vegetable peelings, to obtain zero-waste soup. In this study, the term “zero waste” is used in the context of a valorization approach aimed at utilizing food industry by-products not currently directed to human consumption within a circular economy framework, rather than implying a strictly waste-free process or complete material balance closure. This study also investigated the use of yeast-derived ingredients capable of enhancing umami and kokumi sensations in soup, with the overall goal of producing flavorful broths with desirable nutritional and sensory properties that could be further developed and have potential for industrial application.

## 2. Results and Discussion

### 2.1. Characterization of Zero-Waste Broth

The broth was prepared from poultry carcasses after mechanical meat separation and served as a base for broths made with root vegetable waste from processing lines. Both total and soluble proteins were measured in the basic broth, and the proportions between muscle and connective tissue proteins were calculated based on the amino acid composition of the proteins (Table 1). The content of total and soluble proteins was comparable, whereas the proportions of white muscle tissue and connective tissue proteins differed, amounting to 58.31 g/100 g and 41.69 g/100 g, respectively.

The protein fraction of the basic broth contained all essential amino acids, which must be obtained through the diet because they cannot be synthesized de novo by the human body. Particularly important is the presence of arginine (5.16 g/100 g protein) and histidine (1.41 g/100 g protein), as their endogenous synthesis in infants and children is insufficient, making them conditionally essential during periods of rapid growth and development. Arginine and histidine are primarily found in muscle tissue proteins, whereas connective tissue proteins are rich in proline and amino acids unusual in protein composition, namely hydroxyproline and hydroxylysine. In turn, muscle tissue proteins contain small amounts of leucine, which is present in larger quantities in connective tissue proteins. For this reason, combining these two types of proteins is nutritionally beneficial and increases the nutritional value of the protein in the basic broth.

The peptides and amino acids produced by protein degradation serve as crucial flavor precursors in the formation of meat’s characteristic flavor and its depth. In particular, the relatively high content of glutamic acid (10.27 g/100 g protein) and aspartic acid (7.94 g/100 g protein) is important in the context of umami taste and the enhancement of overall flavor perception [17,18]. Additionally, the high content of glycine (18.63 g/100 g protein), along with alanine (9.81 g/100 g protein) and serine (5.57 g/100 g protein), may contribute to mild sweetness and a flavor-balancing effect. The presence of valine, isoleucine, and phenylalanine, commonly associated with bitter taste, was observed at moderate levels, suggesting their contribution to flavor complexity without necessarily inducing dominant bitterness. Furthermore, sulfur-containing amino acids (cysteine and methionine) are essential for generating characteristic meat-like aromas during thermal processing [18]. Additionally, the high proportion of collagen-derived amino acids (glycine, proline, and hydroxyproline), reflecting the significant contribution of connective tissue proteins, may also influence the mouthfeel and body of the resulting broth [17,18].

**Figure 1:**
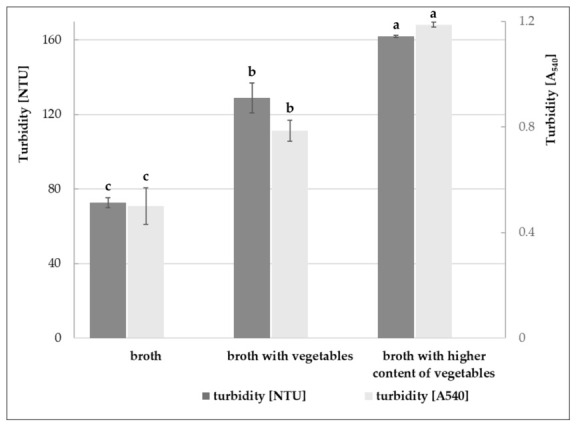
Turbidity of various broths expressed as absorbance at 540 nm and in nephelometric turbidity units (NLUs): broth from poultry carcasses (broth); poultry carcass broth prepared with selected vegetables (broth with vegetables); and poultry carcass broth prepared with higher content of selected vegetables (broth with higher content of vegetables). Means followed by different letters are significantly different (Tukey’s test, *p* < 0.05).

The basic broth served as a base for preparing broths enriched with vegetables (carrots, celery, parsley, and onion), which are waste products from the root vegetable processing line. In both the basic and vegetable-enriched broths, turbidity was measured (Figure 1). This parameter is particularly important in broth-type soups and, to a lesser extent, in multi-ingredient soups. Nevertheless, it remains one of the characteristics that significantly influences consumer preferences. The basic broth was characterized by the lowest turbidity, while the broth with a higher vegetable content had twice the turbidity. It can therefore be concluded that the addition of vegetables caused the formation of an additional colloidal suspension and provided substances acting as emulsifiers. The formulations were further evaluated for their nutritional profiles, with compositional functionality considered more relevant than visual clarity.

Broths with the addition of waste root vegetables contained significant amounts of vitamin K. In the case of both vitamins K1 and K2, almost twice as high concentrations were observed in broths with double the vegetable by-products content (Table 2). In the same broth, a significantly higher concentration of β-carotene, a precursor of vitamin A, was found (1060 µg/100 g) compared to the broth with a single portion of vegetables, while the calculated vitamin A content was 177 µg/100 g.

The antioxidants contained in broths play a significant role in preventing lifestyle diseases. Their primary dietary source is plants, containing polyphenols, carotenoids, tocopherols, and ascorbic acid, among other compounds. Short peptides of animal origin containing the N-terminal amino acid sequence Asp-Arg-Val-Tyr may also exhibit antioxidant activity. For both types of broths, the antioxidant activity was determined using the ABTS and FRAP methods. The antioxidant activity and the ABTS•+ radical scavenging capacity were higher in the broth with double vegetable content (Table 2). This broth also showed the highest ability to reduce iron ions Fe^3+^ to Fe^2+^, as measured by the FRAP method, with a value of 614.5 mg FeSO_4_/L.

Despite its high turbidity, the broth with twice the amount of vegetable waste was used to develop the final broth due to its high vitamin content and antioxidant properties.

### 2.2. Yeast Extracts as a Source of Nucleosides and Vitamins

**Figure 2:**
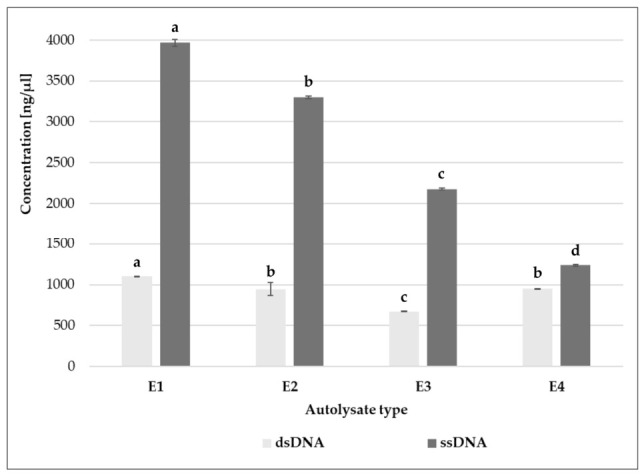
Content of nucleic acids in yeast extracts: E1—autolysate from *Saccharomyces cerevisiae*; E2—*Saccharomyces bayanus*; E3—*Schizosaccharomyces pombe*; E4—*Candida utilis*. Means within the contents of dsDNA and ssDNA followed by different letters are significantly different (Tukey’s test, *p* < 0.05).

Four different yeast species, namely *Saccharomyces cerevisiae* (E1), *Saccharomyces bayanus* (E2), *Schizosaccharomyces pombe* (E3), and *Candida utilis* (E4), were autolyzed and used as sources of nucleoside 5′-phosphate. In yeast autolysates, ssDNA and dsDNA were determined. The highest content of nucleic acids was found in yeast autolysates obtained from *S. cerevisiae* (dsDNA—1101 ng/µL and ssDNA—3965 ng/µL) and *S. bayanus* (dsDNA—945 ng/µL and ssDNA—3301 ng/µL) (Figure 2). These autolysates were subjected to further analyses and treated with RNase (E1/R, E2/R).

The highest concentrations of all analyzed nucleoside 5′-monophosphates were observed in extracts obtained from *S. cerevisiae*. In the extract obtained without treatment (E1) and the extract treated with RNase (E1/R), high contents of 5′-guanosine monophosphate (5′GMP) together with 5′-inosine monophosphate (5′ IMP) were observed (Table 3). Extracts from *S. bayanus* (E2, E2/R) were characterized by trace amounts of 5′GMP and 5′ IMP. Adenosine 5′-monophosphate (5′AMP) was present in the highest amount in the *S. cerevisiae* extract (22.57 μg/mL), and this value was two-fold higher compared to the extract from *S. bayanus*. The use of ribonuclease A did not significantly affect the content of the tested esters. However, in both yeast extracts (E1/R and E2/R), the content of other compounds with a spectrum similar to that of 5′ nucleotides increased.

**Figure 3:**
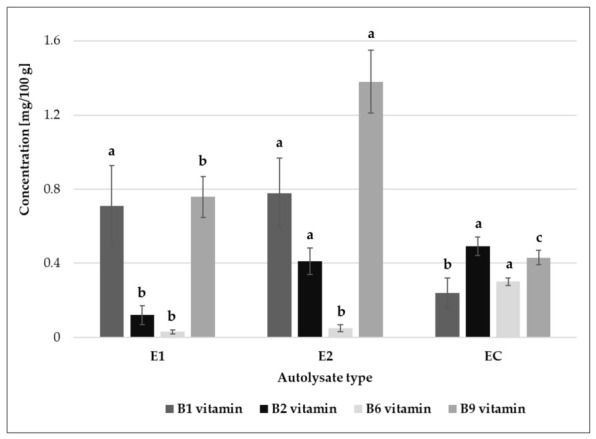
Content of B vitamins in yeast extracts: E1—autolysate from *Saccharomyces cerevisiae*; E2—*Saccharomyces bayanus*; EC—reference yeast extract. Means within the contents of individual vitamins followed by different letters are significantly different (Tukey’s test, *p* < 0.05).

All tested yeast extracts contained B vitamins such as thiamine (B1), riboflavin (B2), pyridoxine (B6), and folacin (B9). Extracts from *S. cerevisiae* and *S. bayanus* were rich sources of thiamine and folacin, while the reference extract was characterized by a higher content of pyridoxine and riboflavin (Figure 3).

The highest antioxidant capacity, measured using the ABTS•+ radical, was observed for yeast extract from *S. cerevisiae* treated with RNase (E1/R). This autolysate was also characterized by the highest degree of ABTS•+ radical neutralization. The reference extract (EC) demonstrated the lowest antioxidant activity and ABTS•+ radical-scavenging capacity (Table 4). In turn, the highest values of antioxidant capacity in relation to the DPPH• radical were obtained for both *S. cerevisiae* yeast autolysates, whether untreated or treated with RNase. These extracts were also characterized by the highest degree of scavenging the DPPH• free radical, above 10%. Additionally, the E1/R autolysate exhibited the highest capacity to reduce iron ions, Fe^3+^ to Fe^2+^, as measured by the FRAP assay. This method yielded a value that was 14% lower for the E1 extract not treated with RNase. The high antioxidant potential of yeast extracts was identified in *S. cerevisiae* and its ability to neutralize free radicals is consistent with the findings of Vieira et al. [19] and Złotek and Świeca [20], who emphasized that yeast-derived extracts could serve as natural antioxidants in functional foods. Therefore, *S. cerevisiae* extract exhibits the most beneficial antioxidant activity and, by neutralizing reactive oxygen species, may help inhibit excessive oxidative stress.

Yeast extracts are a rich source of numerous bioactive compounds, which is why they are used as nutrients and flavorings in food, but also as functional ingredients in functional foods and food additives. In food ingredient lists, they are labeled as “yeast extract” or “natural flavor” [14]. The qualitative and quantitative composition of yeast extracts varies depending on the production method. They primarily consist of proteins, peptides, amino acids (glutamic acid, glycine, alanine, and valine), nucleic acids, as well as B vitamins (B1, B2, B3, B5, B9), minerals (iron, phosphorus, magnesium, and zinc), and carbohydrates [21]. Yeast extracts are used as active flavoring compounds in foods, imparting savory, meaty, and salty flavors. The resulting flavors are influenced by interactions between amino acids, nucleotides, carbohydrates, and peptides present in the extracts, as well as the production of volatile compounds, which are determined by the methods used to prepare the yeast extracts [14]. The flavor-related yeast extract properties should therefore be considered as arising from synergistic interactions among multiple compound classes rather than from individual molecules alone. Accordingly, yeast extracts are used as flavoring ingredients in meat products, soups, broths, sauces, and dressings, as well as in ready-made meals, dietetic and light snacks with reduced fat or carbohydrate content, and plant-based meat alternatives [14,22].

### 2.3. Zero-Waste Broth Enriched with Yeast Extract: Bioactive-Related Properties

Based on the results presented above, a broth was prepared from poultry carcasses obtained after mechanical meat separation and from double the amount of waste products generated during root vegetable processing, to which 2% *v*/*v S. cerevisiae* extract was added. In the obtained broth, the nucleoside content was determined, a consumer hedonic test of the broths was conducted, and the bioactive-related properties were assessed, with a focus on antioxidant activity and vitamin content. Both in the control broth (without the addition of yeast extract) and in the yeast-extract-enriched broth, the presence of 5′-cytidine monophosphate (5′CMP), 5′-guanosine monophosphate (5′GMP), together with 5′-inosine monophosphate (5′IMP), 5′-thymidine monophosphate (5′TMP), and 5′-adenosine monophosphate (5′AMP) was confirmed. However, only trace amounts of uridine-5′-monophosphate (5′UMP) were detected in the analyzed products (Table 5). The addition of yeast extract to the broth significantly affected the 5′AMP content, resulting in an 11% increase compared to the control broth. Furthermore, in both broths, nucleotides esterified at other positions were present alongside nucleosides, with significantly higher amounts in the yeast-extract-enriched broth.

5′GMP and 5′IMP are classified as the nucleotides most responsible for enhancing umami taste [23] and were present in the highest amounts in the analyzed broths. Their contents were ten times higher than those of 5′TMP and four times higher than the amount of 5′CMP (Table 5). The level of added 5′-monophosphate nucleosides required to impart flavor intensity in food ranges from 0.02% to 0.04% [22]. This value was 0.11% in the control broth, whereas the addition of yeast extract increased it to 0.13%. Thus, the addition of yeast autolysate may have contributed to the broth’s flavor intensity, as reflected in the consumer tests, likely through synergistic interactions among nucleotides and other flavor-active compounds.

**Figure 4:**
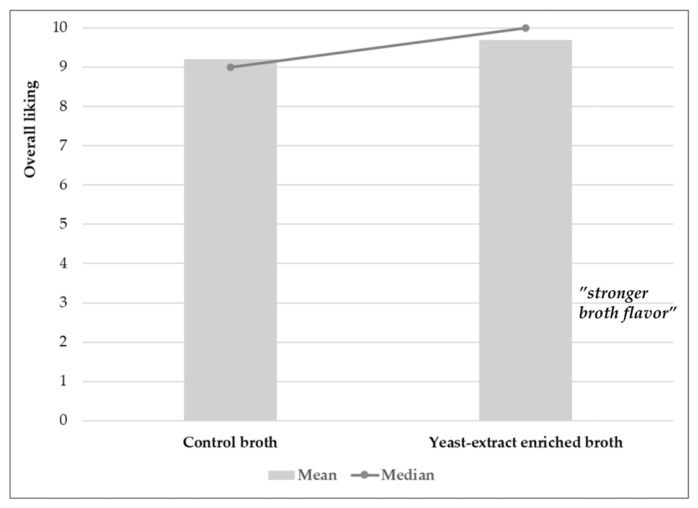
Consumer-based organoleptic evaluation of control broth and yeast-extract-enriched broth.

Based on a preliminary sensory evaluation (n = 10 non-expert assessors), both broths were perceived as desirable. The mean score of the yeast-extract-enriched broth was 9.7 with a median of 10.0, whereas the mean score of the control broth was 9.2 with a median of 9.0 (Figure 4). According to the adopted 10-point hedonic scale, the broth with yeast extract was assessed by panelists as “highly exceptionally desirable,” while the control broth was rated as “exceptionally desirable”. The high acceptability of the control broth may result from the presence of various umami substances, including free amino acids, nucleotides, peptides, organic acids, and their derivatives [24]. However, the addition of yeast autolysate to the tested broth, serving as a flavor potentiator, enhanced the umami taste, which panelists perceived as more brothy and meaty. Nevertheless, the relatively high scores for both broths suggest that the hedonic ratings were close to the upper end of the scale, which may limit the ability to fully interpret the practical significance of the observed differences.

Umami taste is mediated by G protein-coupled receptors (GPCRs), specifically by the T1R1/T1R3 heterodimer. These receptors are expressed not only on the tongue but also in the colon and stomach [25]. The most unique characteristic of umami taste is synergism, whereby the taste of glutamate can be drastically enhanced by 5′-nucleotides, resulting in a markedly stronger intensity of umami [1]. It should also be noted that flavor enhancers are included on the GRAS list and are recognized as safe food additives.

The health benefits associated with the use of yeast extracts rich in 5′-nucleoside monophosphates also result from the possibility of significantly reducing the amount of salt added to food products, according to published data, by as much as half [26]. Moreover, the amount of yeast extract rich in 5′-nucleoside monophosphates required to achieve the desired umami intensity in broth is up to 40 times lower than the amount of monosodium glutamate necessary to elicit a similar sensory effect. 5′IMP, and particularly its salts, not only act as flavor enhancers synergistically with glutamate, but also mitigate an unpleasant vinegar taste and reduce sourness, resulting in a smoother, milder flavor [24,26].

Broth with yeast extract was characterized by a high content of vitamins K and A, as well as B-group vitamins, specifically thiamine (B1), riboflavin (B2), pyridoxine (B6), and folate (B9) (Table 6). The functional levels of vitamin content presented in Table 6 correspond to 15% of the daily reference intakes, in accordance with Regulation (EU) No 1169/2011 [27], which refers to thresholds used in labeling within the framework of nutrition declaration. The amounts of vitamins K, A, B1, and B9 exceed these thresholds in 100 g of broth, whereas vitamins B2 and B6 meet the criteria when calculated per 200 g portion of broth, which is permissible if the package contains only a single portion, as in this case. Thus, the content of all the mentioned vitamins is at a level that allows the use of nutrition claims regarding vitamin content, in accordance with Regulation (EC) No 1924/2006 [28]. Furthermore, the vitamin levels may enable the use of specific authorized health claims related to the physiological functions of these vitamins, as defined in Commission Regulation (EU) No 432/2012 [29], provided that all conditions of use are met.

Bioactive-related properties of both broths also resulted from their high antioxidant activity as measured by in vitro assays (ABTS and FRAP) (Table 5). The antioxidant activity and ABTS•+ radical-scavenging capacity were comparable in both types of broths, with values of 511.20 and 503.68 mg TX/L for antioxidant activity and 70.4% and 68.6% for ABTS•+ radical scavenging for the control broth and the yeast-extract-enriched broth, respectively. The high fat content in the tested broths resulted in the formation of an emulsion with the ethanol used for DPPH reagent preparation, thereby preventing reliable assessment of the antioxidant activity of broths using this method, which represents an important methodological limitation of the assay in such complex food matrices. These limitations of the DPPH method in complex systems are consistent with previously reported methodological constraints, particularly in heterogeneous systems [30].

Higher antioxidant activity of both broths was observed with the FRAP method compared to the TEAC assay, which can be explained by stronger FRAP correlation with total phenolic content and other reducing compounds, such as certain amino acids or carotenoids [31]. Furthermore, significant differences in the antioxidant activity were observed among the analyzed broths, as determined by the FRAP method. The antioxidant capacity of the yeast-extract-enriched broth was 9.5% higher than that of the control broth (Table 5), demonstrating a significant contribution of the added yeast extract to the overall antioxidant activity. This is consistent with the high antioxidant activity of the yeast extract, suggesting its potential contribution as a functional additive.

The broths were prepared from by-products of selected vegetable trimmings, namely carrots, parsley root, onions, and celery, which are sources of various natural antioxidants, including polyphenols, carotenoids, vitamins A and C, tocopherols, phytates, thiocyanates, and isothiocyanates [32]. Moreover, antioxidant activity is a commonly reported property of protein-derived peptides from various meat products and by-products. In chicken meat, an endogenous antioxidant peptide, anserine (N-β-alanyl-1-methyl-L-histidine), is present in high amounts, and its antioxidant activity is primarily attributed to its ability to chelate transition metals [33]. L-lysine and L-histidine have also been reported to exhibit strong ferrous ion-chelating and hydroxyl radical-scavenging activities [34]. Yeast cell lysate contains a high proportion of essential and nonessential amino acids, ribonucleotides, minerals, vitamins, and peptides. Yeast extracts are known for their high antioxidant capabilities, with a major contribution attributed to polysaccharide components of the cell wall, namely mannan and β-glucan [15]. However, the antioxidant properties of yeast extract are also linked to the action of amino acids (histidine, lysine), specific peptides, and glutathione [15,35].

## 3. Materials and Methods

### 3.1. Chemicals and Reagents

Unless otherwise stated, all chemicals and reagents were obtained from Sigma-Aldrich, St. Louis, MO, USA. The chemicals and reagents used in this study included HCOOH (formic acid), C_6_H_5_OH (phenol), HCl (hydrochloric acid), Na_2_S_2_O_5_ (sodium metabisulfite), NaOH (sodium hydroxide), H_2_SO_4_ (sulfuric acid), CuSO_4_ (copper sulfate), K_2_SO_4_ (potassium sulfate), NaCl (sodium chloride), CH_3_OH (methanol), C_2_H_3_N (acetonitrile), ABTS (2,2′-azino-bis(3-ethylbenzothiazoline-6-sulfonic acid diammonium salt; Roche Diagnostics, Mannheim, Germany), K_2_S_2_O_8_ (potassium persulfate), Trolox (6-hydroxy-2,5,7,8-tetramethylchromane-2-carboxylic acid), DPPH (2,2-diphenyl-1-picrylhydrazyl), C_2_H_5_OH (ethanol), TPTZ (2,4,6-tris(2-pyridyl)-1,3,5-triazine, FeCl_3_ × 6 H_2_O (iron(III) chloride hexahydrate), FeSO_4_ × 7 H_2_O (iron(II) sulfate heptahydrate), and KH_2_PO_4_ (potassium dihydrogen phosphate).

### 3.2. Broths Preparation

**Figure 5:**
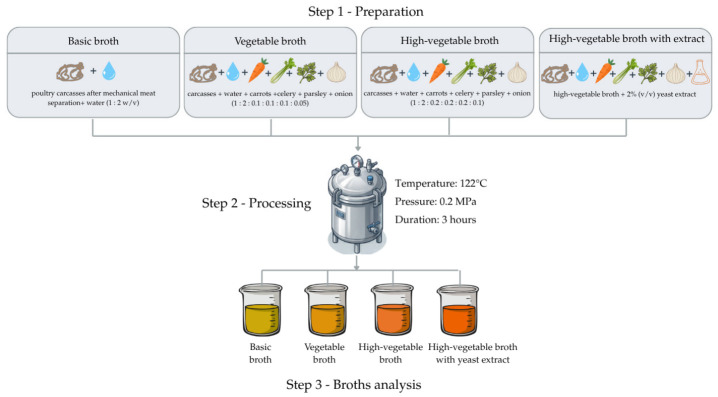
Flow diagram of broth preparation (prepared in Canva).

Four types of poultry broth were prepared: basic, vegetable-enriched with higher vegetable content, and enriched with yeast extract. The basic broth was obtained from poultry carcasses and water (1:2, *w*/*v*) under increased pressure (122 °C, 0.2 MPa) for 3 h in a hermetically sealed pressure vessel. The carcasses after mechanical meat separation were derived from microbiologically tested meat batches that complied with Regulation (EC) No 1441/2007 and met the required microbiological limits [36]. Vegetable broths were prepared analogously using root vegetable by-products. The proportions of carcasses/water/carrots/celery/parsley/onions were 1:2:0.1:0.1:0.1:0.05 for the vegetable broth and 1:2:0.2:0.2:0.2:0.1 for the high-vegetable variant. The yeast-extract-enriched broth was obtained by adding 2% (*v*/*v*) yeast extract, prepared as described in Section 3.7, to the broth with higher vegetable content (Figure 5). All broths were stored at −20 °C until analysis.

### 3.3. Amino Acids Composition of Broths

Amino acid composition was determined according to the Commission Directive 98/64/EC [37]. Briefly, 0.2 g of broth was treated with 0.5 mL of freshly prepared oxidation mixture (H_2_O_2_/formic acid/phenol), incubated at 20–30 °C for 1 h, and cooled prior to use. Samples were oxidized at 0 °C for 16 h to convert methionine and cysteine to methionine sulfone and L-cysteic acid, respectively. After oxidation, Na_2_S_2_O_5_ (0.084 g) was added, followed by 2.5 mL of hydrolysis mixture (HCl/phenol). Acid hydrolysis was carried out at 104 °C for 24 h. Hydrolysates were cooled, neutralized with 40% NaOH, adjusted with citrate buffer (pH 2.2), diluted, and filtered through 0.2 µm nylon filters. Analysis was performed using a Biochrom 30+ amino acid analyzer (Biochrom Ltd., Cambridge, UK) with post-column ninhydrin derivatization. Amino acids were detected after reaction with ninhydrin as colored derivatives at 570 nm, except for proline and hydroxyproline, which were monitored at 440 nm. The injection volume was 20 µL, and the total run time was 90 min.

### 3.4. Determination of Protein Content in Broths

Total nitrogen content was determined using the Kjeldahl method. Briefly, 1 g of broth was digested with 10 mL of concentrated H_2_SO_4_ in the presence of a catalyst (0.5 g CuSO_4_ and 4.5 g K_2_SO_4_) under gradually increasing temperature (200–460 °C) until a clear solution was obtained. After mineralization and cooling, the sample was neutralized, ammonia was distilled, and the solution was titrated with 0.1 mol/L HCl. Protein content was calculated using a conversion factor of 6.25 and expressed per 100 g of broth. Protein content was also determined by the biuret method, according to Liu and Pan [38]. A calibration curve was prepared using a protein standard (a 1:1 mixture of collagen and actin) in the range of 0.2–1.0 mL, adjusted to 1 mL with 0.9% NaCl. Biuret reagent (4 mL) was added, and samples were incubated for 30 min at room temperature. Absorbance was measured at 540 nm against a reagent blank. Broth samples (1 mL) were analyzed analogously, and protein content was calculated from the standard curve and expressed per 100 g of broth.

### 3.5. Turbidity of Broths

Turbidity of the broths was determined by spectrophotometric and nephelometric methods. Absorbance was measured at 540 nm using a V-1200 spectrophotometer (VWR), with water as the reference. Nephelometric measurements were performed using an LTP 6B nephelometer (DR LANGE), and results were expressed in nephelometric turbidity units (NTU). Prior to analysis, a calibration curve was established using appropriate turbidity standards.

### 3.6. Content of Vitamin A and K in Broths

Broth analysis was performed using a UHPLC/DAD/ESI/MS system comprising a UHPLC chromatograph equipped with a diode array detector and a mass spectrometer (LCMS-2020, Shimadzu, Kyoto, Japan). Samples were diluted (0.5 g broth in 10 mL methanol), and 10 µL aliquots were injected onto a Kinetex C18 column (2.1 × 150 mm, 5 µm; Phenomenex, Torrance, CA, USA). The mobile phase consisted of (A) acetonitrile/formic acid (99.9:0.1, *v*/*v*) and (B) methanol, delivered at a flow rate of 0.6 mL/min. The gradient program was as follows: 0–3.0 min, 5% B; 3.0–5.0 min, 5–7% B; 5.0–6.0 min, 7% B; and 6.0–7.0 min, 5% B.

### 3.7. Preparation of Yeast Extracts

**Figure 6:**
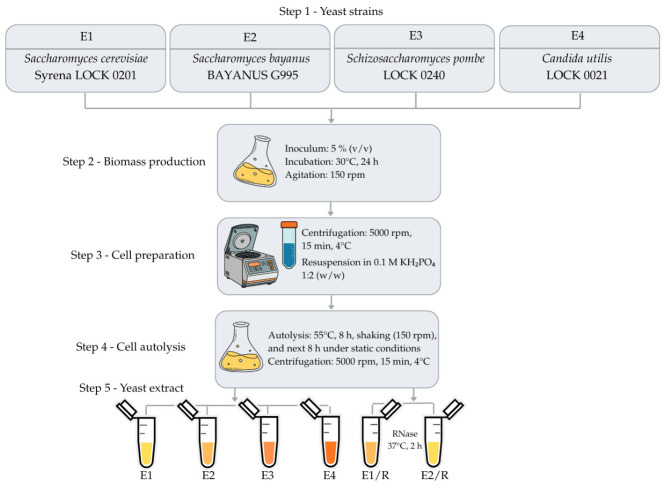
Flow diagram of yeast extracts preparation (prepared in Canva).

Yeast autolysates were prepared from four strains, i.e., three collection strains, *Saccharomyces cerevisiae* Syrena LOCK 0201 (E1), *Schizosaccharomyces pombe* LOCK 0240 (E3), and *Candida utilis* LOCK 0021 (E4), and a commercial strain of *Saccharomyces bayanus* (E2), available as the wine yeast BAYANUS G995 (ARES R.E. Wawrowscy Sp. J., Warsaw, Poland). Yeast biomass was produced by inoculating Sabouraud broth (5% inoculum) and incubating it for 24 h at 30 °C with shaking (150 rpm). The biomass was washed twice with sterile distilled water, centrifuged (5000 rpm, 15 min, 4 °C), and resuspended in 0.1 M KH_2_PO_4_ at a 1:2 (*w*/*w*) ratio. Autolysis was carried out at 55 °C for 8 h with shaking (150 rpm), followed by 8 h under static conditions. Cell debris was removed by centrifugation (5000 rpm, 15 min, 4 °C) (Figure 6). The concentrations of ssDNA and dsDNA in yeast extracts were determined using a NanoPhotometer^®^ Pearl nanospectrophotometer (IMPLEN GmbH, Munich, Germany). Furthermore, autolysates from *S. cerevisiae* Syrena (E1) and *S. bayanus* (E2) were divided into two portions. One portion was treated with RNase A (EURx Ltd., Gdańsk, Poland; 10 mg/100 mL) at 37 °C for 2 h, resulting in RNase-treated samples (E1/R and E2/R, respectively). Autolysates were stored at −20 °C. The reference extract consisted of 0.1% bouillon N, LS, a commercially available savory-soupy yeast extract, derived from genuine brewer’s yeast (Leiber GmbH, Bramsche, Germany).

### 3.8. Determination of Nucleoside 5′-Phosphate Content in Yeast Extracts and Broths

Nucleoside 5′-phosphate content was determined by HPLC-PDA. Samples were filtered through 0.45 μm membrane filters prior to analysis. Chromatographic analyses were performed using a Finnigan Surveyor system equipped with a Finnigan Surveyor PDA Plus detector (Thermo Fisher Scientific Inc., Waltham, MA, USA) controlled by ChromQuest 5.0 software. Separation was carried out on a Spherisorb ODS2 column (250 × 4.6 mm, 5 μm) with a guard column (Waters, Milford, MA, USA). The injection volume was 50 μL, and the flow rate was 0.6 mL/min. The mobile phase consisted of 10 mM KH_2_PO_4_ (pH 5.6) (A) and methanol (B). Gradient elution was applied as follows: 0–25 min, 0–20% B and 25–26 min, 0% B, followed by 14 min re-equilibration, resulting in a total run time of 40 min. Nucleoside 5′-phosphates were quantified using calibration curves for 5′AMP, 5′CMP, 5′GMP, 5′UMP, 5′IMP, and 5′TMP. Nucleosides and nucleotides were identified by UV spectroscopy and expressed as 5′AMP equivalents.

### 3.9. Antioxidant Properties of Broths and Yeast Extracts

The antioxidant activity was evaluated using ABTS•+, DPPH•, and FRAP assays. ABTS•+ radical scavenging was determined according to the modified Trolox equivalent antioxidant capacity (TEAC) method [39]. ABTS radicals were generated by mixing 19.2 mg ABTS (2,2′-azino-bis(3-ethylbenzothiazoline-6-sulfonic acid diammonium salt) and 3.3 mg potassium persulfate in 5 mL water, incubating in the dark for 16 h, and diluting to an absorbance of 0.700 at 734 nm. Samples (30 μL) or Trolox standard solutions (6-hydroxy-2,5,7,8-tetramethylchromane-2-carboxylic acid) were added to 3 mL of ABTS+ solution, incubated 15 min at room temperature, and the absorbance was measured at 734 nm. The percentage of free radical ABTS+ neutralization was determined according to the formula:

ABTS•+ inhibition = ((A_control_ − A_sample_)/A_control_) × 100, (1)

where A_control_ is the absorbance of the ABTS+ solution at 734 nm, and A_sample_ is the absorbance of the test sample at 734 nm.

DPPH• radical scavenging was assessed according to Brand-Williams et al. [40] and Xiao et al. [41] by mixing 50 μL of samples or Trolox standards with 1.95 mL of 24 mg/L DPPH (2,2-diphenyl-1-picrylhydrazyl) in ethanol, incubating for 30 min at room temperature, and measuring absorbance at 515 nm. The percentage of DPPH free radical scavenging was determined using the formula:

% DPPH• inhibition = ((A_control_ − A_sample_)/A_control_) × 100, (2)

where A_control_ is the absorbance of the DPPH• solution at 515 nm, and A_sample_ is the absorbance of the test sample at 515 nm.

The FRAP reagent was prepared by mixing TPTZ (2,4,6-tris(2-pyridyl)-1,3,5-triazine), FeCl_3_ × 6 H_2_O, and phosphate buffer in proportions of 1:1:100. In total, 50 μL of samples or FeSO_4_ × 7 H_2_O standards were mixed with 1.95 mL of the FRAP reagent, incubated for 30 min at 37 °C in the dark, and absorbance was measured at 593 nm. Antioxidant capacity was expressed as mg FeSO_4_ × 7 H_2_O/L.

### 3.10. Determination of B-Group Vitamin Content in Yeast Extracts and Broths

Broth samples or five milligrams of yeast extracts suspended in 1 mL of ultrapure LC–MS-grade water were filtered through a 0.2 µm (17 mm) nylon syringe filter. Analysis was performed using a UHPLC/DAD/ESI/MS system, as described in Section 3.6, with minor modifications. Samples (2 µL) were injected onto a Kinetex C18 column. The mobile phase consisted of (A) acetonitrile/formic acid (99.9:0.1, *v*/*v*) and (B) water at a flow rate of 0.2 mL/min. Gradients were as follows: 0–5.0 min, 0% B; 5.0–5.2 min, 20% B; 5.2–20.5 min, 20% B; and 20.5–20.6 min, 100% B. This was followed by re-equilibration to initial conditions. Mass spectra were acquired in the positive ion mode over the *m*/*z* range of 50–600.

### 3.11. Sensory Evaluation of Broths

The evaluation was conducted with 10 non-expert assessors. Broths were served at 80 °C in a home environment. Panelists evaluated the overall desirability of the broths in triplicate using a 10-point hedonic scale, where 1—extremely undesirable, 2—very undesirable, and 3—undesirable; tolerable quality was assessed as 4—slightly undesirable, 5—neither desirable nor undesirable, and 6—slightly desirable; and desirable quality was assessed as 7—desirable, 8—very desirable, 9—extremely desirable, and 10—very extremely desirable.

### 3.12. Statistical Analysis

All experiments were performed in triplicate, and the results are presented as the mean ± standard deviation. Biological experiments were conducted in triplicate with independent samples, while analytical measurements were performed in triplicate per sample. Statistical differences between means were determined using one-way analysis of variance (ANOVA) followed by Tukey’s post hoc test at a significance level of *p* ≤ 0.05, performed in Microsoft Excel (including the Analysis ToolPak).

## 4. Conclusions

This study presents a promising prototype of a zero-waste broth developed from meat and vegetable processing by-products, with favorable compositional characteristics in terms of nutritional and functional composition. Supplementation with 2% (*v*/*v*) yeast extract may contribute to enhanced umami taste and overall desirability, as indicated by a preliminary sensory evaluation. The broth exhibited high levels of vitamins A (326 µg per 200 g serving), K1 and K2 (27.82 µg per 200 g serving), and B-group vitamins (0.366 mg of B1, 0.296 mg of B2, 0.252 mg of B6, and 1.318 mg of B9 per 200 g serving), reaching concentrations that support nutrition claims and may enable the use of specific authorized health claims related to the physiological functions of these vitamins, in accordance with EU regulations. The bioactive-related properties of the yeast-extract-enriched broth were further supported by its antioxidant activity, as measured by ABTS and FRAP assays (68.6% ABTS•+ radical scavenging, 673.07 mg FeSO_4_/L). The increase in 5′-adenosine monophosphate content due to the addition of a yeast extract may have contributed to enhanced umami intensity. Overall, the study indicates the potential of food industry by-products to be transformed into sustainable products with favorable compositional and preliminary sensory characteristics. These findings highlight the relevance of zero-waste strategies in food production and the importance of sensory optimization for future product development. However, the results should be interpreted as preliminary, and further studies involving larger sensory panels and more comprehensive biological assessments are needed to better elucidate the functional relevance of the developed broth.

## Figures and Tables

**Table 1:** Nutritional composition of the broth from poultry carcasses.

Parameter	Content in Broth
Protein content [g/100 g]	3.13 ± 0.28
Content of soluble proteins [g/100 g]	3.14 ± 0.22
Proportion of white muscle tissue [g/100 g]	58.31 ± 4.35
Proportion of connective tissue proteins [g/100 g]	41.69 ± 4.01
Amino acid composition [g/100 g of proteins]	
Cysteine	0.83 ± 0.06
Aspartic acid	7.94 ± 0.62
Tyrosine	5.22 ± 0.48
Serine	5.57 ± 0.39
Glutamic acid	10.27 ± 0.86
Proline	8.25 ± 0.63
Glycine	18.63 ± 1.02
Alanine	9.81 ± 0.84
Hydroxyproline	3.96 ± 0.27
Hydroxylysine	0.25 ± 0.04
Exogenous amino acids	
Valine	3.91 ± 0.21
Methionine	2.52 ± 0.17
Isoleucine	5.13 ± 0.38
Threonine	2.64 ± 0.19
Phenylalanine	2.58 ± 0.33
Lysine	4.56 ± 0.26
Histidine	1.41 ± 0.09
Arginine	5.16 ± 0.40
Tryptophan	0.55 ± 0.03
Leucine	1.00 ± 0.08
Content of exogenous amino acids [g/100 g of broth]	0.92 ± 0.07

**Table 2:** Vitamin content and antioxidant properties of poultry carcass broth prepared with selected vegetables (broth with vegetables) and poultry carcass broth prepared with a higher content of selected vegetables (broth with higher content of vegetables). Means within the individual parameters followed by different letters are significantly different (Tukey’s test, *p* < 0.05).

Parameter	Broth with Vegetables	Broth with Higher Content of Vegetables
Vitamin K1 content [µg/100 g]	2.020 ± 0.170 ^b^	4.615 ± 0.203 ^a^
Vitamin K2 content [µg/100 g]	4.710 ± 0.396 ^b^	8.505 ± 0.516 ^a^
β-carotene concentration [µg/100 g]	720 ± 34 ^b^	1060 ± 85 ^a^
Calculated vitamin A content [µg/100 g]	120	177
Antioxidant activity		
ABTS [mg TX/L]/% inhibition	426.6 ± 20.6 ^b^/59.7	511.2 ± 22.6 ^a^/70.4
FRAP [mg FeSO4/L]	365.7 ± 19.1 ^b^	614.5 ± 24.8 ^a^

TX—Trolox equivalents.

**Table 3:** Nucleoside content in yeast extracts: E1—autolysate from *Saccharomyces cerevisiae*; E2—autolysate from *Saccharomyces bayanus*; E1/R—autolysate from *Saccharomyces cerevisiae* treated with RNase; E2/R—autolysate from *Saccharomyces bayanus* treated with RNase; EC—reference yeast extract. Means within the contents of individual nucleotides followed by different letters are significantly different (Tukey’s test, *p* < 0.05).

Type of Autolysate	5′CMP	5′UMP	5′GMP and 5′IMP	5′TMP	5′AMP	Other Nucleotides and Nucleosides
	Concentration [µg/mL]					
E1	14.89 ± 1.15 ^a^	not detected	10.47 ± 0.59 ^a^	12.92 ± 0.92 ^a^	22.57 ± 1.11 ^a^	1447.39 ± 9.15 ^b^
E2	14.48 ± 0.52 ^a^	not detected	trace ^d^	9.74 ± 0.73 ^c^	8.21 ± 0.14 ^b^	721.15 ± 5.19 ^d^
E1/R	14.76 ± 1.52 ^a^	not detected	9.91 ± 0.32 ^b^	11.81 ± 0.53 ^b^	3.24 ± 0.08 ^d^	1573.66 ± 17.32 ^a^
E2/R	14.44 ± 1.07 ^a^	not detected	trace ^d^	8.88 ± 0.89 ^c^	7.15 ± 0.32 ^c^	765.14 ± 5.21 ^c^
EC	14.48 ± 0.43 ^a^	0.34 ± 0.01	1.16 ± 0.09 ^c^	8.77 ± 0.36 ^c^	3.13 ± 0.54 ^d^	78.54 ± 1.11 ^e^

**Table 4:** Antioxidant capacities of yeast extracts: E1—autolysate from *Saccharomyces cerevisiae*, E2—autolysate from *Saccharomyces bayanus*; E1/R—autolysate from *Saccharomyces cerevisiae* treated with RNase; E2/R—autolysate from *Saccharomyces bayanus* treated with RNase; EC—reference yeast extract. Means within the methods followed by different letters are significantly different (Tukey’s test, *p* < 0.05).

Type of Autolysate	ABTS		DPPH		FRAP
	mg TX/L	% Inhibition	mg TX/L	% Inhibition	mgFeSO_4_/L
E1	359.9 ± 19.0 ^a,b^	50.4	74.8 ± 8.7 ^a^	10.8	359.3 ± 21.2 ^b^
E2	338.2 ± 18.4 ^a,b^	45.5	60.6 ± 7.8 ^a^	5.1	61.2 ± 7.8 ^d^
E1/R	379.6 ± 19.5 ^a^	50.5	73.1 ± 8.6 ^a^	10.1	416.9 ± 20.4 ^a^
E2/R	326.6 ± 18.1 ^b^	45.0	70.2 ± 8.4 ^a^	8.9	73.5 ± 8.6 ^d^
EC	324.2 ± 17.2 ^b^	45.0	71.1 ± 3.2 ^a^	9.2	254.2 ± 18.6 ^c^

TX—Trolox equivalents.

**Table 5:** Nucleoside content and antioxidant properties in control broth and yeast-extract-enriched broth. Means within the individual parameters followed by different letters are significantly different (Tukey’s test, *p* < 0.05).

Parameter	Control Broth	Yeast-Extract-Enriched Broth
Nucleotide content [µg/mL]		
5′CMP	20.53 ± 0.58 ^a^	21.11 ± 1.11 ^a^
5′UMP	0 ^a^	0 ^a^
5′GMP and 5′IMP	82.90 ± 1.33 ^a^	84.97 ± 0.54 ^a^
5′TMP	8.11 ± 0.54 ^a^	8.13 ± 0.22 ^a^
5′AMP	57.99 ± 0.98 ^b^	65.25 ± 0.72 ^a^
Other nucleotides and nucleosides	970.09 ± 5.31 ^b^	1085.71 ± 7.01 ^a^
Antioxidant activity		
ABTS [mg TX/L]/% inhibition	511.20 ± 22.6 ^a^/70.4	503.68 ± 0.13 ^a^/68.6
FRAP [mg FeSO_4_/L]	614.50 ± 24.8 ^b^	673.07 ± 13.49 ^a^

TX—Trolox equivalents.

**Table 6:** Vitamin content in yeast-extract-enriched broth.

Vitamin	Content Per 100 g of Broth	Content Per 200 g Serving of Broth	Functional Level
Vitamin K1	5.289 ± 0.172 µg	10.578	11.25 µg
Vitamin K2	8.621 ± 0.328 µg	17.242	
Vitamin K1 and K2	13.910 µg	27.82 µg	
β-carotene	981 ± 71 µg	1962 µg	–
Calculated vitamin A concentration	163 µg	326 µg	120 µg
Vitamin B1	0.183 ± 0.019 mg	0.366 mg	0.165 mg
Vitamin B2	0.148 ± 0.017 mg	0.296 mg	0.210 mg
Vitamin B6	0.126 ± 0.024 mg	0.252 mg	0.210 mg
Vitamin B9	0.659 ± 0.074 mg	1.318 mg	0.030 mg

## Data Availability

The original contributions presented in this study are included in the article. Further inquiries can be directed to the corresponding author.
